# Atypical Lesions in Canine Leishmaniosis: Description of New Cases

**DOI:** 10.3390/ani12202784

**Published:** 2022-10-15

**Authors:** María Paz Peris, Paula Ortega-Hernández, Mariano Morales, Juan Antonio Castillo, Bernardino Moreno

**Affiliations:** Animal Pathology Department, Faculty of Veterinary Sciences, University of Zaragoza, 50009 Zaragoza, Spain

**Keywords:** canine leishmaniosis, atypical lesions, histopathological evaluation, immunohistochemistry

## Abstract

**Simple Summary:**

Canine leishmaniosis is a zoonotic disease caused mainly by the protozoan *Leishmania infantum* that is endemic in the Mediterranean basin. The clinical signs and pathological characteristics have been extensively described; however, atypical lesions leading to clinical and pathological misdiagnosis have been sporadically reported. This study presents three cases of dogs experimentally infected with *Leishmania infantum* that showed gross lesions considered atypical of canine leishmaniosis and not previously reported. Macroscopic lesions were observed in the liver, spleen, and peritoneal cavity that corresponded to tan to whitish nodular lesions in all organs and multifocal irregular and linear whitish lesions in one of the livers that mimicked tumors. Microscopically, a marked granulomatous inflammation with a relative scarcity of parasites was observed in all the organs. In conclusion, microscopic and parasitological studies should be performed on atypical lesions observed in *Leishmania*-infected and non-infected dogs, especially in endemic regions. In addition, due to its zoonotic importance, leishmaniosis should be considered when atypical nodular lesions are observed in dogs from non-endemic regions with a history of travel to endemic regions.

**Abstract:**

Pathological characteristics are well described in canine leishmaniosis (CanL). However, atypical lesions that can be confused with other pathologies or trigger unusual clinical signs are sporadically reported. Atypical lesions were observed during routine postmortem examination in three *Leishmania*-infected dogs and samples were taken for histopathological and immunohistochemical studies. Clinical signs, biochemical parameters, level of antibodies, and parasite detection by PCR were also evaluated. Atypical lesions were found in the peritoneal cavity, liver, and spleen. Splenomegalia and hepatomegalia were observed in all dogs. In addition, multifocal dark to white nodules of variable size were observed in the peritoneal cavity, liver, and spleen of one dog and in the spleen of the other two dogs. One dog presented diffuse irregular whitish lines with a threadlike appearance and another an intense fibrotic depression in the intermediate lobe. Microscopically, an intense granulomatous inflammation with abundant macrophages, a variable number of lymphocytes, and a low to moderate number of parasites was observed. This study represents the first description of granulomatous peritonitis associated with *Leishmania* in dogs. It also shows atypical macroscopic expression of hepatitis in CanL. In the absence of an adequate clinical history and laboratory analyses, certain lesions observed in CanL could admit alternative diagnoses.

## 1. Introduction

Canine leishmaniosis (CanL), caused mainly by the protozoan *Leishmania infantum*, is an endemic disease widely distributed throughout the Mediterranean basin. Typical clinical signs can be evident from three months to seven years after infection and are mainly characterized by weight loss, muscle atrophy, skin problems, and, finally, kidney failure [1].

Macroscopic lesions in leishmaniosis typically correspond with clinical signs and are characterized by lymphadenomegaly, muscle atrophy, cutaneous and ocular lesions, onychogryphosis, and nephropathy [1]. Splenic lesions are frequently reported and correspond to marked enlargement, sometimes 2–3 times its normal size, and often accompanied by a dark coloration on the surface. Gross lesions in the liver, however, are rarely reported, although microscopic findings are common. When observed, the liver typically appears symmetrically enlarged and dark brown in color [1,2,3,4,5]. Additionally, hepatitis in canine leishmaniosis is usually subclinical and the pathologic appearance should be considered in the differential diagnosis of the most frequent types of hepatitis in dogs [1]. In addition, the pathologic descriptions can be more complicated in natural cases due to the coexistence of infections with hepatic tropism, such as ehrlichiosis or hepatozoonosis [5].

Atypical gross or microscopic lesions leading to atypical clinical signs have been sporadically reported in CanL. These lesions can often be confused with other pathologies [6,7,8] or be coexisting pathologies [9]. In human leishmaniosis, some lesions or clinical signs have been shown to mimic tumors and differential diagnosis is recommended [10,11,12,13]. In dogs, some *Leishmania*-associated lesions have also been shown to mimic tumors [14]. In addition, *Leishmania* has been detected in some canine tumors, although a clear causal relationship has not been demonstrated [15,16].

In all cases, the microscopic study and demonstration of the parasite in the lesions is definitive for the diagnosis. The histopathological appearance observed in leishmaniosis is dominated by pyogranulomatous to granulomatous inflammation and/or immune-mediated injury, imposed by immune complex deposition or autoantibody generation [17,18]. Immune complex deposition is the mechanism of induction of glomerulonephritis [4]; however, the histiocytic reaction, with a variable number of macrophages, more or less organized, and lymphocytes, plasma cells, and neutrophils, is the main factor responsible for the histological picture observed in lymph nodes, spleen, bone marrow, liver, intestine, bone, genital organs of male dogs, and mucosae [1,19]. The various atypical or rare clinical and pathologic conditions associated with CanL are questioned, since the cause-and-effect relationship has not always been clear [1].

The use of an experimental model with the same dog breed, the same amount of infectious dose, and the same time post-infection for the analyses can clarify some aspects of CanL pathogenesis. In this study, three cases of leishmaniosis with unusual presentations which could lead to alternative diagnosis are presented and a review of atypical lesions is also shown.

## 2. Materials and Methods

### 2.1. Animals and Welfare Statements

Three *L. infantum* experimentally infected beagle dogs derived from a larger study composed of a total of thirty dogs were selected for this evaluation. All animals received regular exercise and social interaction and were housed, maintained, and used for experimentation at optimal conditions: temperature control, ad libitum feeding, procedure refinement, and behavioral enrichment. Furthermore, dogs were periodically examined to determine their health status and to monitor the disease: ~180, ~240, ~300, ~360, and 425 days post-infection (dpi).

As planned in the experimental design, all infected animals were euthanized 425 dpi and a complete necropsy was performed. The three dogs were selected due to the atypical lesions observed in the spleen, liver, and peritoneal cavity during evaluation of gross lesions.

The parasite strain used was MCAN/ES/Z003, which was classified as belonging to genotype A (Maribel Jiménez and Ricardo Molina, personal communication) according to ITS-based genotyping [20]. All efforts were made to minimize suffering. All experimental procedures were approved by the Ethic Committee for Animal Experiments from the University of Zaragoza (Project license PI26/16, date of approval: 18 May 2016).

### 2.2. Serological, Molecular, Biochemical Studies and Clinical Sign Assessment

Prior to euthanasia, serum and lymph node aspirate samples were obtained. In-house Direct Agglutination Test (DAT) and real-time PCR were performed in the Parasitology Laboratory of the Veterinary Faculty of Zaragoza to confirm the experimental infection. Furthermore, clinical signs related to *Leishmania* infection were recorded. DAT antigen and technique protocol were performed as previously described [21,22]. Two-fold dilution series of the sera were made starting at a dilution of 1:100, up to 1:6400. Two independent blind readings were performed by two technicians.

Real-time PCR protocol was performed according to the methods of Peris et al. (2021) [22]. Briefly, popliteal lymph node aspirates (100 µL) genomic DNA were extracted from the samples defrosted from −80 °C using a commercial Speedtools DNA extraction kit (Biotools B&M Labs S.A, Madrid, Spain). A Sybr Green based real time PCR assay was performed in a final volume of 20 µL containing 10 µL of Sybr Green master mix (GoTaq^®^ Hot Start Green Master Mix 2×, Promega, Madison, WI, USA) and 2.5 µL of DNA template. The forward (5′-CCTATTTTACACCAACCCCCAGT-3′) and reverse (5′-GGGTAGGGGCGTTCTGCGAAA-3′) primer concentration was adjusted to 0.4 µmol L^−1^ each.

Biochemical analyses were performed by Laboratorios Albeitar S.L (Zaragoza, Spain) with the Automatic Analyser Gernon Star (RAL, Barcelona, Spain). Determinations and normal values were: bile acids (<10 µmol/L), albumin and globulin ratio (0.7–1.1), alkaline phosphatase (<212 U.I./L in >1 year; <250 U.I./L in 6–12 months; <440 U.I./L in 3–6 months; <530 U.I./L in <3 months), urea (20–40 mg/dL), creatinine (0.5–1.8 mg/dL), aspartate aminotransferase (<60 U.I./L), alanine aminotransferase (<100 U.I./L), and total protein (5.7–7.5 g/dL).

Characteristics of clinical signs of *Leishmania* infection were assessed and their severity recorded. The following signs were evaluated: size of lymph nodes (normal/enlargement); skin involvement (normal/slight scaling and/or alopecia/severe alopecia and/or lesions); weight loss (absence/ moderate <20%/severe >20%); ocular lesions (absence/moderate/severe); onychogryphosis (absence/presence); muscle atrophy (absence/presence); pale mucous membranes (absence/presence); splenomegaly to palpation (absence/presence).

### 2.3. Histopathological and Immunohistochemical Evaluation

Microscopic evaluation was performed to characterize the gross lesions observed. Samples were routinely processed. Briefly, samples were fixed in buffered formalin for 48 h and embedded in paraffin wax. Paraffin blocks were cut at 4 µm and the sections were stained with hematoxylin and eosin (H&E) and examined using light microscopy.

Lymphocytes T, B, macrophages, and parasites were detected by immunohistochemistry as previously described [23]. The following antibodies from Dako (Denmark) were used: polyclonal anti-human CD3 for T-cells (ready-to-use antibody), monoclonal anti-human Clone DAK-Pax5 for B-cells (ready-to-use antibody), and polyclonal anti-human Clone MAC387 for macrophages (1:800 dilution). The technique was performed using a standard protocol with an Autostainer plus (Dako Cytomation, Denmark). A specific rabbit serum raised against *L. infantum* (gift from Dr. Ricardo Molina, Servicio de Inmunología, Instituto de Salud Carlos III, Madrid, Spain) diluted 1:6000 and incubated for one hour, was used as primary antibody for parasite detection.

## 3. Results

### 3.1. Dog 1

#### 3.1.1. Serological, Molecular, and Biochemical Studies and Clinical Sign Assessment

This animal showed low antibody levels against *Leishmania* (1:200 dilution). Parasite DNA was detected by qPCR in the lymph node (Ct = 30.4). Biochemical analyses revealed increased bile acids and urea (16.2 µmol/L and 45 mg/dL, respectively) (Table 1). The other parameters were within normal ranges. The only clinical sign observed in this dog was muscle atrophy.

#### 3.1.2. Gross, Histopathological, and Immunohistochemical Findings

**Figure 1:**
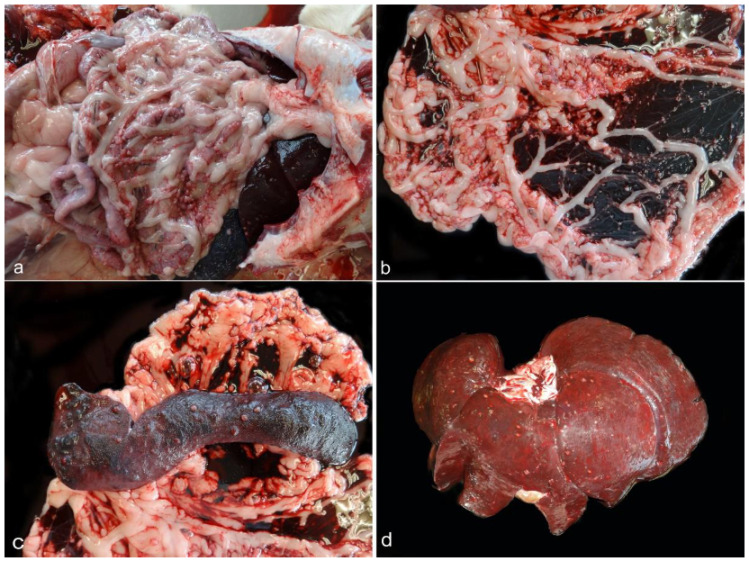
Peritoneum, spleen, and liver from dog 1. Multifocal whitish nodules in the peritoneum, spleen, and liver (**a**). Closer view of lesions in peritoneum (**b**), spleen (**c**), and liver (**d**). Spleen and liver appear enlarged.

Atypical lesions were observed in the peritoneum, spleen, and liver and corresponded with small multifocal red to whitish nodules. In addition, the liver and spleen were moderately enlarged (Figure 1). No significant changes were found in the remaining organs, except for a mild muscular atrophy.

**Figure 2:**
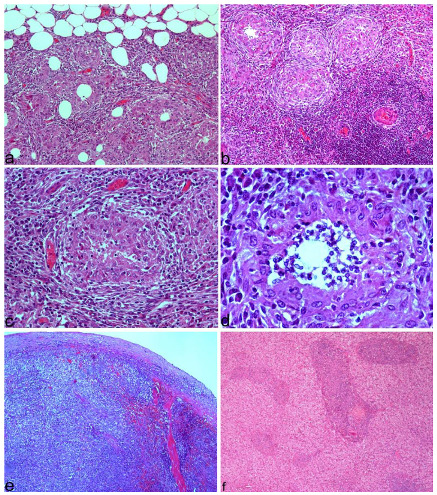
Microscopic findings in dog 1. (**a**) Intense granulomatous peritonitis infiltrating the peritoneal fat, with numerous granulomas and variable number of lymphocytes. H&E; 100×. (**b**) Several granulomas mainly composed of macrophages. Large number of lymphocytes are seen in the lower right area. H&E; 100×. (**c**) Granuloma composed of macrophages and few lymphocytes. H&E; 200×. (**d**) Piogranuloma with neutrophils in the center. H&E; 400×. (**e**) Marked granulomatous splenitis. H&E; 50×. (**f**) Granulomatous hepatitis with multifocal variable-sized granulomas. H&E; 50×.

Microscopically, the peritoneal lesions corresponded to severe granulomatous peritonitis, with numerous granulomas surrounded by a variable number of lymphocytes and plasma cells that infiltrated and faded the peritoneal fat (Figure 2a). Most of the granulomas were formed solely by macrophages (Figure 2b); however, in some of them a variable number of neutrophils appeared, mainly in the center (Figure 2d). Macrophages also appeared scattered throughout the inflammatory reaction mixed with lymphocytes and plasma cells (Figure 2c). The lesions in the spleen and liver were similar to those described in the peritoneum (Figure 2e,f).

**Figure 3:**
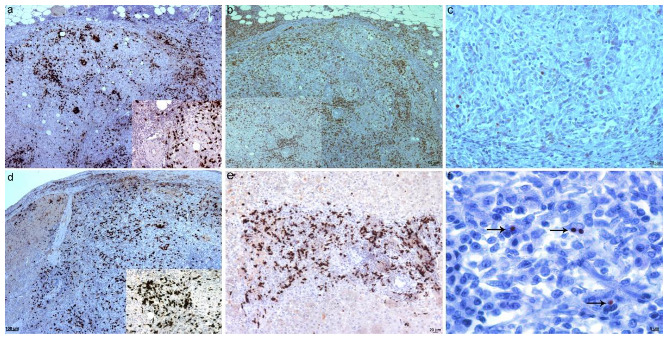
Immunohistochemical findings in dog 1. (**a**) Peritonitis with numerous macrophages forming granulomas and scattered throughout the peritoneum, 50×. Inset: detail of granulomas infiltrated by numerous macrophages (polyclonal antibody against MAC387 antigen), 200×. (**b**) Peritonitis with numerous T lymphocytes irregularly distributed between granulomas, 50×. Inset: detail of T lymphocytes surrounding and infiltrating granulomas (polyclonal antibody against CD3), 100×. (**c**) Scattered B lymphocytes in area with intense inflammation (monoclonal antibody against Pax5 antigen), 200×. (**d**) Granulomatous splenitis with numerous macrophages. 50×. Inset: detail of granulomas infiltrated with macrophages, 200×. (**e**) Granulomatous hepatitis with numerous macrophages (polyclonal antibody against MAC387 antigen), 100×. (**f**) Amastigote forms in inflammatory reaction. Arrows mark brown immunostained parasites (polyclonal antibody against *Leishmania*), 630×.

Immunohistochemistry demonstrated the presence of numerous macrophages forming granulomas throughout the peritoneum (Figure 3a) and lymphocytes, the majority of which were T lymphocytes (Figure 3b). T lymphocytes were located mainly around the granulomas, although some also infiltrated some granulomas. The presence of B lymphocytes was scarce (Figure 3c). Similarly, immunohistochemistry revealed granulomatous splenitis (Figure 3d) and hepatitis (Figure 3e). *Leishmania* was observed to have a low incidence and was seen mainly within scattered macrophages or outside cells, but not in granulomas (Figure 3f).

### 3.2. Dog 2

#### 3.2.1. Serological, Molecular, and Biochemical Studies and Clinical Sign Assessment

This animal showed high antibody levels against *Leishmania* (1:1600 dilution). Parasite DNA was detected by qPCR in the lymph node (Ct = 29.4). Biochemical analyses showed variations in ALT (112 UI/l), urea (43 mg/dl), total proteins (7.9 g/dl), and alb/glob ratio (0.5) (Table 2). Clinical signs assessment in this dog revealed muscle atrophy presence and slight conjunctivitis.

#### 3.2.2. Gross, Histopathological, and Immunohistochemical Findings

**Figure 4:**
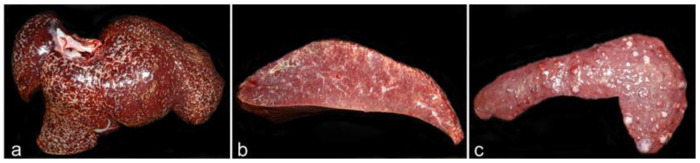
Liver and spleen from dog 2. (**a**) Enlarged liver with irregular whitish lines with diffuse distribution. (**b**) Section of the liver with whitish lesions throughout the parenchyma. (**c**) Enlarged spleen with multifocal whitish nodular lesions.

In this dog, the liver was enlarged and presented irregular whitish lines with a threadlike appearance and a diffuse distribution (Figure 4a,b). The spleen was also enlarged and presented with multifocal whitish nodules (Figure 4c).

**Figure 5:**
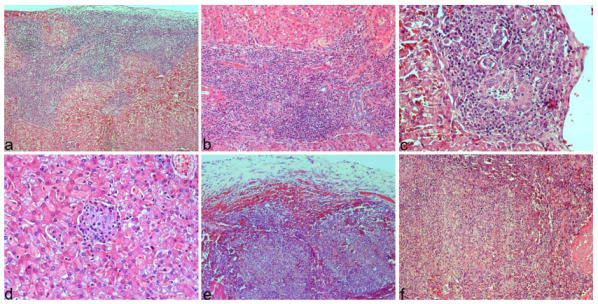
Microscopic findings in the liver and spleen in dog 2. (**a**) Granulomatous hepatitis mainly located in subcapsular area and portal tracts. H&E; 50×. (**b**) Marked inflammatory infiltrate in portal areas. H&E; 100×. (**c**) Portal tract with one well-formed granuloma and numerous macrophages not organized and mixed with lymphocytes. H&E; 200×. (**d**) Small well-defined granuloma in the parenchyma. H&E; 100×. (**e**) Granulomatous splenitis with numerous macrophages below the capsule. H&E; 100×. (**f**) Granulomatous splenitis with numerous macrophages that do not form organized granulomas. H&E; 100×.

Histologically, the liver lesions corresponded to a severe granulomatous hepatitis with numerous lymphocytes, located mainly in the portal areas, and which spread through the parenchyma bridging several portal spaces. The inflammatory infiltration was also very intense under the capsule in some areas (Figure 5a). The hepatitis was composed of an intense infiltrate of macrophages, lymphocytes, and plasma cells (Figure 5b,c). In areas with severe inflammation, macrophages formed poorly defined granulomas, often infiltrated by lymphocytes (Figure 5c). Scattered, well-defined, and predominantly small multifocal granulomas were also observed throughout the parenchyma (Figure 5d). The splenic lesions corresponded to a severe granulomatous splenitis with multifocal granulomas and numerous macrophages scattered among numerous lymphocytes (Figure 5e,f).

**Figure 6:**
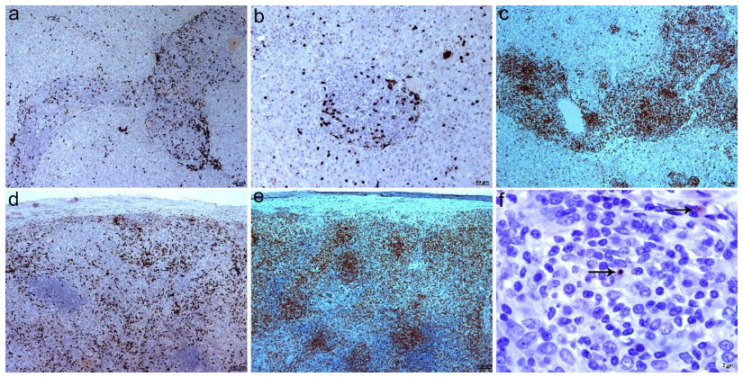
Immunohistochemical findings in dog 2. (**a**) Numerous macrophages infiltrate mainly portal areas and spread through the parenchyma bridging the portal areas (polyclonal antibody against MAC387 antigen), 200×. (**b**) Detail of macrophages forming a granuloma and scattered throughout the parenchyma (polyclonal antibody against MAC387 antigen), 400×. (**c**) Numerous T lymphocytes infiltrating portal areas and spread through the parenchyma bridging the portal areas (polyclonal antibody against CD3), 50×. (**d**) Granulomatous splenitis with numerous macrophages near the capsule (polyclonal antibody against CD3), 100×. (**e**) Granulomatous splenitis with numerous T lymphocytes forming aggregates near the capsule (polyclonal antibody against CD3), 100×. (**f**) Scattered amastigotes in the spleen, associated with macrophages not organized in granulomas. Arrows mark brown immunostained parasites (polyclonal antibody against *Leishmania*), 630×.

Immunohistochemistry revealed the presence of numerous macrophages, mainly scattered among lymphocytes and less frequently forming granulomas (Figure 6a,b). As described in dog 1, numerous T lymphocytes were also present, mixed with macrophages and infiltrating some granulomas (Figure 6c–e). *Leishmania* was scarcely detected in the lesions and mostly associated with macrophages outside the granulomas (Figure 6f).

### 3.3. Dog 3

#### 3.3.1. Serological, Molecular, and Biochemical Studies and Clinical Sign Assessment

This animal showed high antibody levels against *Leishmania* (1:800 dilution) and qPCR also detected parasite DNA in lymph node (Ct = 28.9). Biochemical analyses showed slight variations in ALT (122 UI/L) and urea (45 mg/dL) (Table 3). Evaluation of clinical signs revealed only moderate conjunctivitis.

#### 3.3.2. Gross and Histopathological Findings

**Figure 7:**
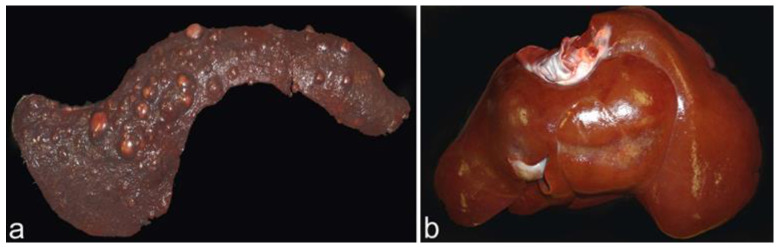
Liver and spleen from dog 3. (**a**) Mildly enlarged spleen with multifocal tan to whitish nodular lesions. (**b**) Enlarged liver with a marked depression, of fibrotic appearance, in the medial lobe.

The gross findings observed in this dog were enlargement of the liver and spleen with a marked grayish depression with fibrotic appearance in the medial lobe of the liver and multifocal tan to whitish nodules in the spleen (Figure 7).

**Figure 8:**
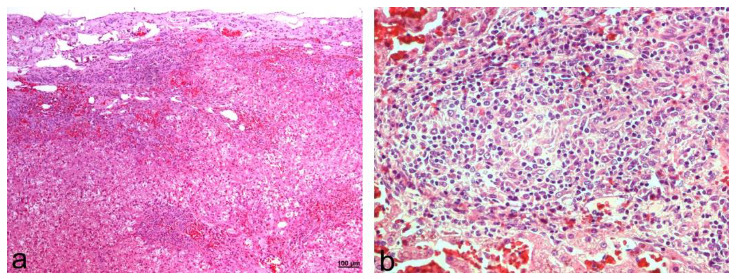
Microscopic findings in dog 3. (**a**) Granulomatous hepatitis, located mainly in the portal and subcapsular areas, with collapsed parenchyma, capsular fibrosis, evident blood vessels, and marked fatty degeneration of hepatocytes. H&E; 50×. (**b**) Granulomatous hepatitis with macrophages forming a poorly organized central granuloma, mixed with lymphocytes. H&E; 200×.

Histological evaluation revealed severe granulomatous hepatitis with collapse of hepatocytes and loss in superficial areas (Figure 8a,b). Evident blood vessels and marked fibrosis were observed under the Glisson´s capsule in this area. Hepatocytes in these areas and all around the liver showed severe fatty degeneration. The spleen lesions were similar to those in dog 2. Immunohistochemistry was not performed on this dog; however, it was suspected to be similar to dog 2 due to the similar pathology, with low quantities of parasites.

## 4. Discussion

Pathological characteristics are well defined in canine leishmaniosis with lymphadenomegaly, splenomegaly, cutaneous and ocular lesions, and nephropathies as prominent macroscopic lesions [1]. Microscopically, the lesions correspond with a granulomatous to pyogranulomatous inflammation with the presence of parasites. Sporadically, atypical lesions in classic organs mimicking other pathologies or lesions in atypical locations have been reported [6,14,24]. Furthermore, in some of these cases, macroscopic lesions are the only finding observed and diagnosis can be difficult, preventing adequate treatment. Only microscopic studies of atypical lesions can demonstrate their association with *Leishmania* infection. In some cases, atypical lesions have only been demonstrated after studying atypical clinical signs and adequate response to treatment after diagnosis [8,25,26]. Additionally, lesions in natural cases in endemic regions can be modulated by concurrent infections [27].

Atypical lesions have been described in various organs of dogs infected with *Leishmania* [6]. In the digestive system, atypical lesions have been reported more frequently on the tongue. Tongue lesions have been shown as single lesions in some dogs and can be nodular [6,7,28] or ulcerative [29], which represents a diagnostic challenge. *Leishmania* has also been observed in the colon of diarrheal dogs with a variety of lesions including ulcerative colitis [30], histiocytic colitis, and lymphoplasmacytic colitis [31]. *Leishmania*-associated duodenitis is also an atypical presentation and has been a cause of diarrhea in some dogs [8,32]. Cutaneous lesions are common in canine leishmaniosis and are typically characterized by exfoliative dermatitis and alopecia. Atypical and much less common lesions that lead to misdiagnosis are nodular dermatitis and sterile pustular dermatitis [6]. Atypical lesions in the musculoskeletal, articular, cardiovascular, respiratory, or nervous systems have also been described [1,6,8,25,33]. In the present study, atypical lesions were observed in the peritoneal cavity, liver, and spleen.

Small focal light-colored nodular lesions can be observed in various organs of *Leishmania*-infected dogs and represent one of the greatest diagnostic challenges in CanL, especially when they appear isolated [1,14,34]. This is especially relevant in non-endemic regions [14]. In the present study, multifocal nodules of variable size were observed in the peritoneal cavity, liver, and spleen of one dog and in the spleen of two other dogs. The peritoneal lesions suggested chronic peritonitis of various etiologies; however, lesions mainly resembled pyogranulomatous or granulomatous peritonitis. In dogs, these lesions are mainly observed in eumycotic mycetoma or caused by zygomicetes in which nodular lesions are observed in the omentum and mesenteries [35]. Less often, nodular peritonitis has been caused by *Mycobacterium microti, Actinomyces* spp., or *Nocardia* spp. [35,36] or associated with larval stages of parasites such as *Mesocestoides* or *Spirometra* [37]. Microscopic and immunohistochemical studies in our case finally revealed granulomatous peritonitis associated with *Leishmania* and ruled out bacterial, fungal, and some parasitic etiologies. Furthermore, the experimental protocol used in this study made it possible to rule out others causes, especially those of infectious etiology. The peritoneal lesions described in this study represent the first description of peritonitis in CanL. Although a sclerosing peritonitis has been reported in a dog infected with *Leishmania*, its association with *Leishmania* was not finally demonstrated, and it was considered a coexisting lesion [9]. Sclerosing peritonitis, moreover, is very different from that observed in our case. The former is characterized by severe peritoneal fibrosis that encapsulates abdominal organs [38], while nodular lesions were observed in our case. The peritonitis observed in the present study was probably caused by the extension of superficial lesions in the liver and spleen. Alternatively, a marked systemic cellular response induced by the parasite may have also triggered the inflammatory response in the peritoneum.

Furthermore, the peritoneal lesions resembled peritoneal tumors, either primary such as mesothelioma, or secondary such as carcinomatosis or sarcomatosis [38,39]. Mesothelioma is uncommon in dogs, accounting for less than 0.1% of neoplasms, and typically affects adult dogs. There are a variety of histologic lesions, but nodular lesions are frequently observed macroscopically. Secondary tumors in the peritoneal cavity typically manifest as nodular lesions and are most often due to spread of tumors of abdominal organs [38]. As mentioned above, nodular lesions in the peritoneal cavity can be of infectious or non-infectious etiology. Interestingly, carcinomatosis has been confused with *Mesocestoides*-associated lesions [37]. Regardless of the origin of the peritoneal nodular lesion, microscopic examination is always recommended [38]. In some cases, even techniques such as immunohistochemistry are necessary to demonstrate the cellular origin [38]. Mesotheliomas are positive for both cytokeratins and vimentin, while carcinomatosis lesions are only positive for cytokeratins, and sarcomatosis only for vimentin. In our study, the histological study ruled out a neoplasm.

Splenic lesions are common in CanL and typically correspond with splenomegaly. Splenic parasite-associated nodular lesions have rarely been reported in dogs [1,40]. Freitas et al. (2016) found macroscopic black nodules in a *Leishmania*-infected dog that microscopically corresponded to nodular hyperplasia [40]. In human medicine, nodular lesions in the spleen have also been exceptionally associated with *Leishmania* [41,42]. However, nodular lesions in the spleen are very common in dogs, especially older dogs, and may or may not accompany splenomegaly. Therefore, a differential diagnosis is always recommended. Benign lesions correspond mainly to nodular hyperplasia, which can be purely lymphoid or complex hematomas, hemangiomas, and extramedullary hematopoiesis. Malignant lesions are mainly hemangiosarcoma, histiocytic sarcoma, lymphomas, or other sarcomas [4,43,44,45]. Due to this large number of differential diagnoses of splenic lesions in dogs, a microscopic study is always recommended in dogs infected with *Leishmania*, especially in natural cases in which other infections may coexist, such as some hemoparasites or protozoa that can induce splenomegaly and lymphoid hyperplasia [43]. In the present study, all dogs presented with whitish nodules that mainly resembled hematopoietic tumors such as lymphoma or histiocytic sarcoma [35,46]. Lymphoma is one of the most common tumors in dogs and can affect various organs. In the spleen, lymphoma can manifest as splenomegalia or as nodular lesions [46]. Histiocytic sarcomas are included within the broad spectrum of canine histiocytic diseases and can be localized to multiple sites due to their origin in interstitial dendritic cells. They can show a variety of macroscopic appearances, and whitish nodules are typically observed in the spleen [43]. The gross lesions observed in the present case closely resembled splenic histiocytic sarcoma; however, the microscopic and immunohistochemical studies ruled out this tumor or lymphoma and demonstrated a severe granulomatous splenitis and the presence of *Leishmania*.

Gross lesions in the liver of dogs infected with *Leishmania* are far less common than splenic lesions and correspond mainly to hepatomegaly. Occasionally, light-colored focal nodular lesions can be observed in the liver [1,34]. However, microscopic lesions are very common and mainly characterized by granulomatous hepatitis [1]. In our case, hepatomegaly was observed in all dogs; however, the lesion found in dog 2 has not been described in CanL. This lesion was consistent with the severe subcapsular hepatitis and bridging of portal tracts observed microscopically and could suggest chronic hepatitis of various etiologies or hematopoietic neoplasms. Hepatitis is frequent in dogs and can be associated with infectious, toxic, or autoimmune causes [47,48]. It can be acute or chronic and its macroscopic appearance is usually nonspecific; however, the aspect observed in our case has not been found. Granulomatous hepatitis is a type of chronic hepatitis in dogs and can be caused by pathogens such as *Mycobacterium* sp. [48], mycoses, or some parasites [48], or by pathogens with atypical location in the liver such as *Leptospira* sp. [48,49] or *Angiostrongylus vasorum* [50]. It can also be observed in chronic cases of copper excess [48]. In the present study, the experimental procedure allowed us to rule out infectious agents and the microscopic study to rule out tumors or other pathologies. The collapsed liver observed in dog 3 was also atypical. Hepatic parenchymal depressions are usually associated with traumatic lesions; however, in our case it was suspected to be associated with the severe inflammation and fatty degeneration of hepatocytes below the capsule. The fibrotic aspect suggested a chronic lesion.

Like splenic nodular lesions, hepatic masses are common in dogs and may be associated with benign or malignant lesions [47,51,52]. Benign lesions are usually nodular and can be isolated or multifocal [53]. Tumors, whether benign or malignant, are usually nodular with white nodules typically seen in hematopoietic tumors. However, some tumors can acquire a diffuse pattern, such as lymphomas, histiocytic sarcoma, and mast cell tumors. In these cases, enlargement of the liver is usually seen. In this study, hepatomegaly was observed in all dogs and nodular lesions in only one dog. In this dog, the simultaneous presence of similar lesions in the spleen and peritoneum also suggested a tumor, either primary or metastatic. In the dog, metastatic tumors in the liver are more common than primary tumors, and lymphoma appears to be the most frequent [53]. The microscopic study of the nodular liver lesions ruled out a tumor and revealed a granulomatous hepatitis associated with *Leishmania*. In dog 2, the gross appearance also suggested marked cell infiltration that might correspond with a tumor and histologic examination again showed hepatitis. Interestingly, the microscopic distribution of cells in hematopoietic tumors is different, with portal and central vein involvement in most lymphomas and sinusoidal location in myeloid tumors [53]. In severe lymphomas, bridges of tumor cells can be observed between the portal and central areas. The distribution of the lesions observed in our cases was similar to that observed in lymphoma, although the lesions corresponded to granulomatous hepatitis.

Immunohistochemistry is recommended for the diagnosis of leishmaniosis, especially in lesions with few parasites. In our case, this technique confirmed the presence of the parasite in all the lesions, although the parasite load was generally low. The low presence of *Leishmania* observed in these lesions is compatible with the intensity of the granulomatous reaction, which is usually associated with a greater capacity to eliminate the parasite [4]. Also interesting is the relationship reported between *Leishmania* and tumors, and immunohistochemistry can be a very useful technique. In addition to the possibility of mimicking tumors, as previously described [14], *Leishmania* has been detected in tumor cells of certain neoplasms, both in the dog [16,54,55] and in humans [10,13]. However, a causal relationship has not yet been demonstrated [54], although it suggests the ability of *Leishmania* to spread and infect different types of cells [16]. It has been suggested that, in endemic areas, tumors should be a differential diagnosis and their possible association is worth investigating. Chronic inflammation has been shown to induce some tumors [44] and *Leishmania* typically triggers very chronic inflammation. In our study, macroscopic lesions could suggest a tumor; however, no neoplastic cells were observed after histopathological examination.

Biochemical analyses associated with liver dysfunction revealed altered liver enzymes, but no clinical disease was observed. A significant increase in bile acids was found in dog 1 compared to dog 2. Furthermore, ALT was slightly altered and slight hypoalbuminemia was observed in Animal 2. These changes probably indicate slow disease progression, rather than an extensive and acute inflammation. However, normal ALT activity does not necessarily imply that the liver disease is inactive [3]. Finally, the increase in bile acids suggests hepatic dysfunction. These changes were, however, nonspecific, as they can be observed in other chronic hepatopathies [56]. In *Leishmania*-infected dogs, the most significant biochemical changes are hyperglobulinemia and altered renal parameters.

## 5. Conclusions

In conclusion, this study adds new atypical manifestations of *Leishmania* infection in dogs. It represents the first description of granulomatous peritonitis, indicating that leishmaniosis should be included in the differential diagnosis of peritonitis in dogs. It also showed that the nodular lesions occasionally seen in the spleen and liver can mimic tumors. Furthermore, it shows that severe hepatitis can occasionally manifest macroscopically as white irregular linear lesions. To the best of our knowledge, this lesion has not been reported to date. Finally, it reinforces the need to perform microscopic examination and use immunohistochemistry in any lesion found in dogs infected by *Leishmania*.

## Figures and Tables

**Table 1:** Dog 1 biochemical parameters.

Bile Acids	Creatinine	Alkaline Phospatase	AST	ALT	Urea	Serum Proteins	Alb/Glob Ratio
16.2 µmol/L	1.1 mg/dL	25 UI/L	33 UI/L	51 UI/L	45 mg/dL	6.9 g/dL	1.1

**Table 2:** Dog 2 biochemical parameters.

Bile Acids	Creatinine	Alkaline Phospatase	AST	ALT	Urea	Serum Proteins	Alb/Glob Ratio
9.6 µmol/L	1.0 mg/dL	151 UI/L	32 UI/L	112 UI/L	43 mg/dL	7.9 g/dL	0.5

**Table 3:** Dog 3 biochemical parameters.

Bile Acids	Creatinine	Alkaline Phospatase	AST	ALT	Urea	Serum Proteins	Alb/Glob Ratio
8.4 µmol/L	0.8 mg/dL	140 UI/L	45 UI/L	122 UI/L	45 mg/dL	7.1 g/dL	0.9

## Data Availability

Not applicable.
